# Researches on the Natural History of Death

**Published:** 1850-10

**Authors:** 


*Researches on the Natural History of Death.* By Bennet Dowler, M. D., of New Orleans. 8vo. pp. 22.

Various circumstances have interfered with our intention to acknowledge the receipt of Dr. Dowler's characteristic essay at an earlier date. We have further to regret that similar interruptions will compel us to confine the present notice of his views within narrower limits than the importance of the topic may demand.

No one who reflects that in the midst of life we are in death, can fail to take a deep interest in every intelligent and independent discussion of the momentous question to which the doctor's paper is devoted in the present instance. We gladly undertook the examination of these "Researches" therefore, and would still more gladly introduce them to our readers sufficiently *in extenso* to enable them to speak as effectually for themselves to others as they have to us, if such a course were practicable in a brief review of an article which itself is entirely too brief and occasional in character to admit of lengthened critical analysis.

Without attempting "a formal monograph on death," or "even an outline of the several branches of the natural history of death," he has given to his readers, in the course of twenty-two pages, (written, according to his own account, "in fragments, one portion having been in the printer's hands while another remained to be written,") an amount of information and highly suggestive speculation that is not only curious in itself, but entitled to attention as the offering of a physiological observer of established experience and sagacity. Indeed, with all due deference to our author's other inclinations, or perchance more important occupations, we think his readers would have been much more thankful, and much better able to do justice to his work, had he chosen to spend a little more time and pains upon its elaboration. Grateful as we are for the favor of his lucubrations on the subject, their very richness in fact and illustration has filled us with regret that the formal monograph disclaimed, or, at any rate, that "an outline" were not the actual result. The "preludes" of death, he tells us at the outset, "its characteristics, general and special, its progress, its prognostics, and its pathological anatomy, will be omitted, not for want of facts, but because the facts I have collected on these subjects are too numerous and unwieldy for the narrow limits to which this paper must be restricted. The pathological anatomy of death, the changes due to the agony *itself*, its immediate antecedents and effects, physical and physiological, including the order in which functions cease, and tissues die, together with the resulting anatomical alteration, being subjects of great importance, and deserving of the fullest investigation, cannot be disposed of in a summary manner. The period intervening between the agony and the usual time of post-mortem examination is rich in facts which have been too much neglected." All this is doubtless as true as words can make it, while it tells our trouble at the present writing just as clearly. It is the old story over again—so much to do, and so little time to do it in. Hence our tears. We hear of the great treasure which "still so near us, yet beyond us lies;" we are graciously allowed a glimpse of it, and see and taste just enough of its mysterious wealth to make us hunger and cry for more with all our might.

The leading object of the author seems to be to notice, "more as an experimenter than as a critic," "the criteria of the certainty of death, particularly the tests of death as set forth by the recent proceedings of the Academy of Sciences of Paris, in the award of the Manni prize." He endeavors to show that the means of distinguishing real from apparent death, proposed by the successful aspirant, M. Bouchut, and sustained by the Academy, are not by any means as conclusive as the French savans would have us to believe. But in thus destroying the fair fabric of our Gallic compeers, erected on the discovery of the test by auscultation of the heart's action, Dr. Dowler hastens to calm the fears of the most timid of his readers with a triple source of consolation.

Not content with assuring us in the strongest manner, what we fully believe, that *bona fide* premature interments are rare almost to an infinite degree, and that the ordinary signs are all-sufficient, he proposes a test of death himself, which he considers more available, as well as more reliable, than that of M. Bouchut. This substitute for auscultation, is not, we are told, the result of mere closet speculation, but "is founded on numerous prolonged experiments, probably one thousand, made directly on several hundred bodies." For the reasons already given in regard to the unavoidable omission of many interesting topics, nothing is said, in this discussion, "of the peculiar physiognomy of the dead, the lividity and cadaveric injection of dependent parts, the flattening of the tissues that sustain the weight of the body, and the other signs of death of minor importance," among which we may enumerate loss of elasticity, opacity of the fingers, the peculiar inward flexure of the thumbs, the expulsion of alimentary substances from the mouth, cessation of capillary circulation, and loss of susceptibility to the action on the skin of external irritants.

Following our author in his own way and order, we have first to call attention to a few remarks respecting contractility and two or three other so-called phenomena of life alone. He repeats, what he has often said elsewhere, that "contractility, animal heat, capillary circulation, and the like, do not prove the body to be alive in the popular, legal, and utilitarian sense." "As to what is commonly called life, the body may be wholly deprived of it without necessarily losing muscular contractility or muscular life." "Life is an aggregation of vital phenomena, or of means and ends; death is a destruction or alteration of these means and ends: all those vital phenomena which remain after the extinction of all the useful means and ends of life do not really constitute life in its ordinary acceptation or essential condition, implying thinking, feeling, willing, and acting." "Contractility, in its isolated character, does not prove that life is present in its ordinary or utilitarian sense; nor is rigidity, as is generally assumed, absolutely incompatible with the contractile power. These forces are neither incompatible nor identical, much less are they connected as cause and effect."

In regard to *rigidity* as a criterion of death, we may as well place in this connection the addendum on that subject with which the tract concludes:

"Rigidity, as a criterion of death, is inconvenient in practice, as it may be tardy in its appearance, and occasionally absent, or of very short duration. Hence its verification requires the constant presence of the physician; otherwise, it might appear and disappear during a short absence from the corpse, throwing doubt on the certainty of the death, and causing delay in the burial. Still, however, it is a sign of great value, and the manner or order in which it disappears is highly characteristic. The suppleness or relaxation of the muscles very generally take place first in those parts that were the first invaded by the rigor mortis, as, for example, in the neck. Rigidity is liable to other objections: it may be simulated; it may originate mesmerically, and convulsively."

Cadaveric rigidity, however, is more easily overcome than the simulated, and when overcome it does not return. It somewhat resembles also the stiffness of congelation, but need not be confounded with it. Rigidity in the corpse is best recognised, as our author intimates, by its successive phenomena in progress, and above all by the peculiar relaxation which succeeds it. Relaxation following rigidity, in the opinion of good authority, is one of the most confirmatory signs.

In continuation of the pamphlet, we next have a very entertaining and instructive historical sketch of the inquiry, especially in its relations to the institution of the prize under the auspices of the Parisian Academy. This, together with a digression by the way respecting early post-mortems in warm climates, and sundry other matters, we would be glad to lay before our readers in the doctor's own words, if the space had been allowed us.

After some five pages of these curiosities of resurrectionary experience, he returns to the Manni prize. This was placed, by a royal decree, at the disposal of the French Academy in 1837, and had been founded "with the hope," for obvious reasons, "of discovering some other satisfactory sign *anterior to that of decomposition.* According to M. Bouchut, the successful candidate, the "certain signs of death are *immediate or remote.* The first consists in 1, the prolonged absence of the sounds of the heart; 2, the simultaneous relaxation of the sphincters; and 3, the sinking of the globe of the eye, with loss of transparency of the cornea. The first of these, alone, is regarded by the committee as conclusive. The *remote* signs are: 1, cadaveric rigidity; 2, the absence of muscular contractility under the influence of galvanism; and 3, putrefaction."

Dr. Dowler's first objection to the principal or cardiac test, that its application must be limited, "because there are but few good stethoscopists among good practitioners, and in the best hands, certainty is often not attainable," is certainly not a strong one against its scientific value. For granting that the "actual state of auscultation" is so lamentably low, the distinction in this case to be ascertained, is not between certain sounds of the heart under different states of action, but between the presence and absence of any kind of sound whatever—a distinction which can be made by any one possessed of hearing, whether that hearing be previously sharpened by exercise or not. It would be hard to imagine any sound in the thorax, especially in the cardiac region, of a corpse, that could be mistaken for that of the pulsation of the heart; at all events we are not aware of any yet on record. The next objection is much more to the purpose, although even there the learned experimentalist, in our humble opinion, is running a little too far in his comparative physiology, in spite of the excuse he gives when he cites the hearts of alligators in his illustration of the same organ in man. But let us hear him in his own words:

"The Academy, and M. Bouchut, take for *granted*, that which may not be *true, and which is the very thing to be proved!* Who has proved that the heart, like the pulse, like every other organ, may not fall into *temporary quiescence or inaction*? May not the heart itself suffer apparent not real death, as all analogies drawn from other muscular organs teach? The sphincters, uterus, intestines, and stomach, the respiratory, lingual, ocular and locomotive muscles may be palsied, inactive, apparently dead for a time. It is a downright begging of the question, to assume that the heart *cannot itself fall into this very state of apparent death.* The natural history of the movements of the heart, indicates the probability of a temporary suspension of action; at one time it gives 200, at another 8 or 10 strokes in a minute; it intermits, or is irregular. In cholera it is probably cramped in some cases; temporarily quiescent in others. Moreover, it has, in common with other muscles, a kind of life of its *own;* it is not the known sole criterion of general life. Comparative physiology shows that an animal may live hours without the heart, and the heart for days* without the body. An alligator's heart will act with regularity for many hours, perhaps for days, after having been cut out of the body, and emptied of its blood. Let an alligator thus deprived of its heart, be roasted; return its heart, and apply the stethoscope, and then the dead will afford this certain sign of life! The commission of the Academy cannot object to this argument, because they themselves experimented on the inferior animals in testing M. Bouchut's claims: as for myself, I have shown, in my published papers, that I attach less importance to comparative physiology, as the interpreter of human physiology, than systematic writers do themselves. If I take their own point of departure, they can require nothing more.

"M. Dumas, author of the Article *Cœur*, in the Dictionnaire d'Histoire Naturelle, quotes Bacon, Haller, and Diemerbroeck, who state, that in man the heart may be removed without suddenly extinguishing life, and that men have looked about, spoken and prayed after having lost their hearts by the executioner; though M. Dumas does not vouch for the truth of these statements. (iv. 289.)

"Moreover, has any one asserted, much less has any one proved, that the action of the heart is always appreciable; that it never can be so feeble as to escape observation, remote as it is from sight, and even from the hearing? To say with the Academy, that a *prolonged absence* of cardiac sounds, is an absolute proof of death, is vague and unsatisfactory; *prolonged absence* of animal heat, or of respiration, would equally prove the reality of death, not to mention other tests, as rigidity, &c.

The very object contemplated by the Manni prize, is to dispense with this prolongation; for if a prolongation be necessary, there is an infinitely better test; one absolutely certain, the characteristics of which all know as well as the Academy, namely, putrefaction, and which ought not to have entered into the enumeration of M. Bouchut at all. Had M. Bouchut adopted as the criterion of death, the prolonged absence of respiration, the rest had been equally, nay more certain, and, withal, of easier application, than the uncertainties of auscultation. "If the respiration," says Dr. Paris, "be suspended only *five minutes*, we may conclude that life is fled forever. Of all the acts of animal life, this is by far the most essential. Breath and life are properly considered in the Scriptures as convertible terms; and the synonyme, as far as we know, prevails in every language."

*This I cannot vouch for as an observation made by myself, but Dr. Lindsay, of this city, has seen the separated heart of the alligator still in action, on the second day after its removal from the body, which I fully believe, though I have never watched the heart more than 6 to 9 hours continuously after separation.

"As M. Rayer, the reporter, and his coadjutors of the committee, regard this auscultatory test as the principal feature of M. Bouchut's essay, it is proper to look a little further into this matter. M. Rayer and the commissioners made some experiments on the human subject, and on animals; from which they conclude, with M. Bouchut, that "the absence of pulsation of the heart for *five minutes* leaves no doubt of the cessation of life;" but how many experiments they made does not appear: let us suppose fifty. Now from the very nature of the case, this is but a negation; a *non sequitur;* for it might have happened, that all, or a portion of the next fifty cases would have revived after a *temporary cessation* of the heart's action. Suppose the commission had tried the non-respiratory test for the same period, namely *five minutes*, would they have found any revivals? Would they not have been able to draw a conclusion equally certain?"

In conclusion of this branch of the argument, we may as well annex another "*addendum*" of the author, which he has inserted as an afterthought at the end of his pamphlet. It is no doubt well known to many of our readers.

"Mr. John Hunter, the celebrated surgeon, long before death, had according to his own statement, (confirmed by his medical attendants, Sir George Baker, and Doctors William Hunter, Huck, Saunders and Fordyce,) an alarming spasmodic attack, in *which the heart's action entirely ceased for three quarters of an hour.* 'This curious fact in physiology, says his biographer, has never been satisfactorily explained.' Mr. Hunter's intellectual powers remained unimpaired. He sustained his respiration by forced, or rather voluntary efforts."

Next we are treated to the "Shaksperian criteria of death." These "will probably not fail once in a thousand years, if ever." We omit the verse, giving only the paraphase in prose: "The circulation ceases; the body cools; the breathing ends; an ashy paleness replaces the natural hues; the cornea grows dim and relaxes; rigidity prevails. These signs may safely challenge, for certainty, all those of the stethoscopes of the whole academy."

The ordinary tests of respiration, such as the motion of the chest, ascertained by sight, and the action of the breath itself in misting the looking glass and agitating the suspended feather, although good tests, may be fortified, according to our author, "by other respiratory phenomena which have been little noticed in this connection, by poets or physiologists, namely—the *peculiar progressive, or rather retrogressive manner by which respiration*

*recedes from the lungs to the trachea*, from the latter to the larynx, from the larynx to the mouth, and from the mouth to the very lips; this is characteristic, nay, conclusive, if I may judge, of true death, though I do not know that it has ever been regarded at all. It is indeed very different from the cessation of breath in cases of suspended animation, or apparent death, in hysteria, catalepsy, croup, convulsion, stagnation, fainting and the like. The manner of the cessation of respiration, though indescribable to the inexperienced, is very peculiar, and is the earliest absolute sign of real death. The præ-mortem signs are very conclusive." This is all, doubtless, very true, but we need hardly stop to say why their applicability as tests must be limited to a comparatively small number of cases. M. Bouchut's second great sign of death, sustained by the academy, the relaxation of the sphincters, is totally discredited by Dr. Dowler. In denying its authority, the latter speaks from "an experience in this particular line that probably has never been equalled—an experience incidental to several years' experiments on animal temperature—a single experiment often costing several hours, during whieh thermometers have been repeatedly passed within the sphincters." "The latter," continues the Doctor, "with very few exceptions, contract strongly after death. Relaxation of the sphincters is not a post mortem, but an ante or præ-mortem phenomenon. It happens sometimes as a disease, and is not a fatal symptom."

The third and last sign relied on by M. Bouchut and the Academy is not more satisfactory to our investigator. He insists with great reason that it fails in uniformity, whether we study its condition before or after death. We know that it is sometimes a well marked ante-mortem symptom, and that at other times it is absent for some time after vitality has ceased. So much for the Academy and its chosen apostle. We have seen the different fragments of their really noble monument to the philanthropy of the Italian abbé, and their own scientific enterprise, one by one assailed, if not actually demolished. We turn now with renewed interest to the single pillar which our worthy friend has undertaken to plant in opposition to the more imposing structure of his brother savans. Let him speak to us once more in his own language:

"I propose the thermometer as a means of testing death, possessing, as it does, superior certainty over the stethoscope. The latter method takes for granted, that in apparent death, the heart's action continues; that it cannot be for a time suspended, and that its action can always be heard! The very analogies of apparent or temporary death seem to oppose or contradict these assumptions. The analogies and the positive facts known of animal temperature, teach that, during life, the body is not heated and cooled like inert matter. Place two or three thermometers in the arm-pits—in the bend of the arm, (the fore-arm being flexed,)—in the mouth and within the sphincters, to ascertain the heat of the surface, and of the centres, (the rectum is the best and most accessible centre.) The application of the thermometer requires no skill, and is open to the inspection of all, and is a test for all the warm blooded animals, at least for man. While the auscultatory test takes for granted that there can be no temporary inaction of the heart, and that all its motions can be heard; the thermometrical test takes nothing for granted without the most indubitable proof. Its great axiom is that man, in his living state, maintains an uniform temperature, independent of the surrounding media, while a dead man, like other inert matter, has no independence of this kind, but steadily responds to, and is governed by, calorific conditions altogether physical—heating and being heated, receiving and radiating caloric. This is not the result of speculation, but of prolonged and varied experimental research.

"The refrigeration of the body before death, in cholera, congestion, and the like, is not physical refrigeration, responding to the calorific condition of the surrounding media; it is a morbid, or physiological caloricity, which, for a time, augments or continues stationary after death, until it shall be replaced by physical refrigeration, as its phenomenal history clearly shows.

"The facts which I have published concerning post mortem caloricity, do not invalidate this thermometrical test; for soon, or late the physical refrigeration must take place. I may here add, that the speculative opinion which prevails among those who do not take the trouble to make experiments, namely, that these calorific movements are the effects of putrefaction, is wholly unfounded (so far as it regards the human subject;) how much soever it may be countenanced by certain analogies derived from other inert matter. The calorific, and the putrefactive periods, so far from coinciding, antagonize each other, so long as the heat is not in accordance with the ordinary physical laws of caloric. The point of coincidence and equilibrium, is really the point of putrefaction, unless the circumstances be of an extraordinary character, such as involve the freezing point, or that of torrefaction. But the predomination of the invariable law of physical refrigeration, is a criterion always attainable, and may be proved, as to its times, distances, and velocities, by arithmetical calculation; ascertain the temperatures of the media, and of the heated body; the velocity of the refrigeration will be proportioned to the times and distances, and will proceed from the surface to the centre, until the equilibrium be attained. The only objection that lies against this rule relates to calorific conditions, where the differences between the heated body and the media are very slight; but this is of no importance in practice, because there is always a marked difference between the average temperature of the air in the shade, and that of a living, or recently dead person."

In thus bringing forward his substitute for the Bouchut test, Dr. Dowler seems to have covered the ground in behalf of its superior certainty and availability, and to have met the practical objections to it well enough to satisfy an attentive reader previously familiar with the subject. It is a matter of regret, however, that in the introduction of so important an adaptation of a striking physiological truth, he has not taken the time and trouble, albeit at the expense of other matter, to explain his views, and especially his practical deductions, with more fulness and precision. It would not have been hard for him, with his idea of its extreme simplicity, to bring the application of the test he advocates, at least as much within the reach of general readers, as M. Rayer and Bouchut have brought the test which he rejects. The philosophy of vital heat, and of its præ-mortem and post-mortem changes in different parts of the body, under various internal and external influences, including the best mode of appreciating their condition and their results, is not quite so easily comprended, even by the trained observer. It is certainly not less puzzling than the simple axiom of the incompatibility of the slightest sound of cardiac pulsation with actual death, and *vice versa.* There is no doubt about the change of temperature after death; and he who runs may read how low the thermometer has gone in any given case and in the course of a given time. But it is not every one who can so calculate "the times and distances" as to tell how low the thermometer ought to go, or how rapidly it ought to fall.

Dr. Dowler is not singular in his denial of certainty to the inaction, or inappreciable action, of the heart as an evidence of death. The merely negative assertions of the Academy, founded on a limited experience, which determined five minutes to be the longest possible suspension of the cardiac action during life, had been nullified by the positive data of Braschet and others (supposing them to be worthy of respect) before the scruples of their American antagonist had been presented to the public. M. Braschet informs us in his protest,* that he had repeatedly restored the vitality of infants, in whom no pulsation whatever could be discovered, from fifteen to thirty minutes, although his mode of sub-diaphragmatic exploration (i. e. the application of one or two fingers under the ribs, and between the diaphragm and liver directly under the pericardium,) practised by himself extensively for thirty years, is, in his opinion, as effectual as the nicest auscultation. This writer furnishes his readers with two recent examples, in point. The first was that of a child, who had revived after *twenty minutes'* insufflation, although during that time no trace of pulsation could be heard or felt. The other was that of a man æt. 33, whose heart presented no contraction that could be detected during at least eight minutes, although the ear was applied again and again. *Twenty minutes* after the suspension of its action a slight contraction was perceived in the heart; its pulsations then became regular, and the patient opened his eyes. "Boiling water was thrown upon the limbs" of the poor man, and every possible external stimulant was applied, doubtless including artificial heat; so that, in the latitude of New Orleans, he might have been stripped for the tender mercies of the ice-box or dissecting table, or perchance enclosed in his coffin for immediate interment, almost before refrigeration could have had a chance to display its progress on the most susceptible thermometer. A medical friend of ours, in the course of a steamboat voyage up the Mississippi last summer, was present at the digging of a grave on shore, intended for a fellow-passenger, who was dying of cholera. The poor creature had gone on with his apparent refrigeration, while his provident undertakers were at work upon his contemplated lodging. He disappointed them at last, however; for when they were ready he was not, inasmuch as, although the heat had left his body, the breath was still in it. We do not know that such *contretemps* as this ever happen in New Orleans to embarrass the preparation for the premature examinations which are common in that city. Sure we are, however, that we should have little faith in any sliding scale of refrigeration, however central in its appli-

* Gazette des Hopitaux, No. 135.

cation, while such a complexity of horrors were menacing our closing hours. With the scalpel already bared on the one hand, a yawning grave on the other, and no other admissible alternatives but the ice-box or putrefaction, we would rather a thousand times encounter the worst exhalations of a dead-house than trust the disposal of a friend's remains to the decision of the best thermometer that ever was made.

After all, the difficulty is vastly more apparent than real. At the present day, especially, is it infinitely more serious in theory than in practice. Premature interments and marvellous revivals appear frequently enough among the items of the newspapers; but in the grave-yards themselves, at all events in the British, German, and American burying-grounds, such accidents are utterly unknown. It is otherwise among the French, if we are to listen to the startling representations of their numerous writers on the subject. There is every reason, however, to accord with Dr. Dowler and the London Quarterly Review* in estimating the statistics and various romantic stories of Bruhier, Louis, Thierry, Nysten, Fontenelle, and others, as so many draughts on the credulity of a wonder-loving public.

The signs of death, properly so called, should be studied under different aspects. First, when the opportunity allows, they should be watched in their progress before, as well as directly after, the final change; secondly, in their succession after death; and, lastly, in their assemblage as a whole. Many of them are important individually, but it is in the last named general view that they become, in spite of adverse theory, sufficiently conclusive for all practical purposes.

Having disposed to his satisfaction of the prominent points in the discussion of the criteria of certain death, our author continues his "Researches" in some seven pages of highly interesting miscellaneous matter, embodying a variety of curiosities of medical and philosophical experience, chiefly relating to the "*agony*" of death. This portion of the pamphlet we are compelled to pass without further comment than to recommend it to the attention of all who have time to avail themselves of the inexhaustible ingenuity and reading of its author.

To return for a moment to the question of the difference be-

* No. clxx, Oct., 1839.

tween real and apparent death, we cannot do better than to conclude with the closing survey of a recent French reviewer in the *Gazette Medicale.* We present it as much for the summary it gives as for the precept it enjoins. "Experience," says he, "has shown the insufficiency of each of these signs, with one exception—*putrefaction.* The absence of respiration and circulation, the absence of contractility and sensibility, general loss of heat, the hippocratic face, the cold sweat spreading over the body, cadaveric discoloration, relaxation of the sphincters, loss of elasticity, the flattening of the soft parts on which the body rests, the softness and flaccidity of the eyes, [the loss of catoptric reflection too, and opacity and dryness of the cornea,] the opacity of the fingers, [and, we may add, the peculiar inward bending of the thumbs,] cadaveric rigidity, the expulsion of alimentary substances from the mouth; all these signs combined or isolated, may present themselves in an individual suffering only from apparent death. Doubtless their complete co-existence, except in case of actual death, is extremely rare. Doubtless, too, some of these signs have so discouraging a signification, even separately, that their presence is equivalent to a sentence of death. Still, in so grave a matter the very rarest exception possesses an infinite value. We will go farther and say, that it ought even to weigh as much as the rule itself in governing the conduct of the physician and attendants. Here, if ever, is the axiom, *Melius anceps quam nullum,* to be applied. Were the chances of success only one in a thousand, one in ten thousand, or a hundred thousand, every precaution, every measure likely in a remote degree to realize such success, becomes a bounden duty."

